# Drug-resistant tuberculosis in subjects included in the Second National Survey on Antituberculosis Drug Resistance in Porto Alegre, Brazil*, **

**DOI:** 10.1590/S1806-37132014000200009

**Published:** 2014

**Authors:** Vania Celina Dezoti Micheletti, José da Silva Moreira, Marta Osório Ribeiro, Afranio Lineu Kritski, José Ueleres Braga

**Affiliations:** Federal University of Rio Grande do Sul, Porto Alegre, Brazil; Department of Pulmonology, Federal University of Rio Grande do Sul, Porto Alegre, Brazil; Institute of Microbiology, Federal University of Rio de Janeiro, Rio de Janeiro, Brazil; Department of Pulmonology, Federal University of Rio de Janeiro School of Medicine, Rio de Janeiro, Brazil; Department of Collective Health, Rio de Janeiro State University; and Public Health Researcher. Oswaldo Cruz Foundation, Rio de Janeiro, Brazil

**Keywords:** Tuberculosis/diagnosis, Drug resistance, HIV

## Abstract

**OBJECTIVE:**

To describe the prevalence of multidrug-resistant tuberculosis (MDR-TB) among tuberculosis patients in a major Brazilian city, evaluated via the Second National Survey on Antituberculosis Drug Resistance, as well as the social, demographic, and clinical characteristics of those patients.

**METHODS:**

Clinical samples were collected from tuberculosis patients seen between 2006 to 2007 at three hospitals and five primary health care clinics participating in the survey in the city of Porto Alegre, Brazil. The samples were subjected to drug susceptibility testing. The species of mycobacteria was confirmed using biochemical methods.

**RESULTS:**

Of the 299 patients included, 221 (73.9%) were men and 77 (27.3%) had a history of tuberculosis. The mean age was 36 years. Of the 252 patients who underwent HIV testing, 66 (26.2%) tested positive. The prevalence of MDR-TB in the sample as a whole was 4.7% (95% CI: 2.3-7.1), whereas it was 2.2% (95% CI: 0.3-4.2) among the new cases of tuberculosis and 12.0% (95% CI: 4.5-19.5) among the patients with a history of tuberculosis treatment. The multivariate analysis showed that a history of tuberculosis and a longer time to diagnosis were both associated with MDR-TB.

**CONCLUSIONS:**

If our results are corroborated by other studies conducted in Brazil, a history of tuberculosis treatment and a longer time to diagnosis could be used as predictors of MDR-TB.

## Introduction

There was a recrudescence of tuberculosis in the late 1980s, which led the World Health Organization (WHO) to declare it a public health emergency in 1993.^(^ ^1^ ^)^ In early 1994, the WHO also initiated the Global Project on Anti-Tuberculosis Drug Resistance Surveillance, in collaboration with the International Union against Tuberculosis and Lung Disease (IUATLD).^(^ ^2^ ^)^ Between 1994 and 1999, the WHO and the IUATLD compiled drug resistance data from surveys carried out in 58 countries.^(^ ^3^ ^)^ They found that the mean prevalence of primary multidrug-resistant tuberculosis (MDR-TB), in patients with no history of tuberculosis treatment was 1.0% (range, 0-14.1%), and that the mean prevalence of acquired MDR-TB was 9.3% (range, 0-48.2%).^(^ ^3^ ^)^

Studies conducted between 2002 and 2006, collectively involving 90,000 patients in 81 countries, demonstrated an increase in the estimated prevalence of drug-resistant tuberculosis (DR-TB).^(^ ^4^ ^,^ ^5^ ^)^ In 2005, there were 500,000 new cases of MDR-TB worldwide, corresponding to 5% of the total number of cases of tuberculosis. In that same year, the prevalence of primary MDR-TB was 2.9% (range, 2.2-3.6%), whereas that of acquired MDR-TB was 15.3% (range, 9.6-21.1%), respectively.^(^ ^4^ ^)^

In 2006, cases of extensively drug-resistant tuberculosis (XDR-TB) were reported in South Africa, mostly in HIV-infected hospitalized patients. By 2009, cases of XDR-TB had been reported in various other regions of the world. ^(^ ^6^ ^)^ Research also showed that death rates were higher in countries with an elevated prevalence of tuberculosis/HIV co-infection (which included cases of MDR-TB or XDR-TB in HIV-infected individuals), underscoring the need for effective interventions for the prevention and treatment of infection with resistant strains of *Mycobacterium tuberculosis*.^(^ ^7^ ^)^

In Brazil, reductions in incidence and mortality rates suggest that the tuberculosis situation has improved over the last ten years. However, in certain metropolitan regions of the country, there has been no improvement at all.^(^ ^2^ ^)^ For instance, in the southern Brazilian city of Porto Alegre, the incidence of tuberculosis increased from 97/100,000 population to 116/100,000 population between 2001 and 2009, 30% of all tuberculosis cases reported for the city being diagnosed in hospitals. That increase was accompanied by a high prevalence of tuberculosis/HIV co-infection, a decrease in the tuberculosis cure rate (from 69% to 65% of all treated cases) and an increase in the rate of default from treatment (from 15% to 20%).^(^ ^8^ ^)^

In 1996, the First National Survey on Antituberculosis Drug Resistance was conducted in Brazil.^(^ ^9^ ^)^ Participants were recruited from 13 health care facilities throughout the country, and approximately 6,000 strains of M. tuberculosis were identified.^(^ ^9^ ^)^ The prevalence rates of primary and acquired MDR-TB were 1.1% and 7.9%, respectively.^(^ ^10^ ^)^ However, the survey did not assess the prevalence of HIV infection and was limited to patients treated at primary health care clinics.^(^ ^10^ ^)^ In southern Brazil, the prevalence rates of primary and acquired MDR-TB (0.8% and 5.8%, respectively) were lower than the nationwide prevalence.^(^ ^10^ ^)^ Since then, no other epidemiological (population-based) studies of antituberculosis drug resistance have been conducted in any of the major cities of southern Brazil. Therefore, the present study aimed to characterize the prevalence of DR-TB and MDR-TB in the city of Porto Alegre, where the efficacy of tuberculosis control programs has decreased significantly in recent years. The present study was also aimed at identifying the prevalence of HIV infection and any demographic or clinical characteristics associated with antituberculosis drug resistance in a population recruited from primary health care clinics and hospitals.

## Methods

The data analyzed in the present study were collected in the city of Porto Alegre as part of the Second National Survey on Antituberculosis Drug Resistance, conducted between 2006 and 2007. Between March of 2006 and December of 2007, patients were recruited from five primary health care clinics and three public hospitals. All patients provided sputum samples for smear microscopy and mycobacterial culture. The samples were also tested for resistance to rifampin, streptomycin, ethambutol, and isoniazid. However, due to the poor reproducibility of tests for resistance to streptomycin and ethambutol, those results were not considered in the present study.

On the basis of the results of the bacteriological examination, we defined DR-TB as resistance to any antituberculosis drug and MDR-TB as resistance to (at least) the combination of isoniazid and rifampin. The presence of organisms resistant to one or more drugs in patients with no history of tuberculosis treatment, or with prior treatments lasting one month or less, was classified as primary drug resistance. The presence of resistant microorganisms in patients with a history of tuberculosis treatments lasting over a month was classified as acquired drug resistance.

Given the differences in the expected prevalence of rifampin resistance in new patients (primary resistance) and re-treated patients (acquired resistance), minimum sample sizes were calculated for these two groups. These calculations were performed using a proportional-to-population-size cluster sampling method, taking into account the size of the tuberculosis diagnostic facilities and consequently the number of patients admitted for diagnosis and treatment at each health care facility.^(^ ^11^ ^,^ ^12^ ^)^

Participants were recruited from five primary health care clinics (Modelo; Navegantes; Institute for Childhood Protection and Assistance; Vila dos Comerciários; and Sanatório), as well as from three hospitals (the Nossa Senhora da Conceição Hospital of Porto Alegre; the Sanatório Partenon Hospital; and the Porto Alegre Hospital de Clínicas of the Federal University of Rio Grande do Sul School of Medicine), all located in the city of Porto Alegre. All patients who visited any of these health care centers during the recruitment period and were suspected of having pulmonary tuberculosis were eligible for participation. Suspected pulmonary tuberculosis was defined as the presence of respiratory symptoms or clinical or radiological signs of tuberculosis, as per the Brazilian National Guidelines for the Control of Tuberculosis.^(^ ^13^ ^)^ Mycobacterial cultures were carried out for all clinical samples, regardless of the sputum smear test results.

Eligible patients were included in the study if they met one of the two following criteria: being classified as a new case (no history of tuberculosis treatment) with culture-positive pulmonary tuberculosis (regardless of smear test results); and having a history of tuberculosis treatment (relapse or history of default from tuberculosis treatment), presenting with culture-positive pulmonary tuberculosis, or having used antituberculosis drugs in the 30 days prior to survey participation and sputum sample collection. We applied the following exclusion criteria: being under 18 years of age; being pregnant; and having negative culture results (regardless of the smear microscopy results) or no drug susceptibility testing (DST) results (i.e., DST not carried out in accordance with the Brazilian National Guidelines for the Control of Tuberculosis).^(^ ^13^ ^)^ Sputum samples were collected prior to the beginning of treatment. Patients were not required to consent to HIV testing in order to participate in the survey.

Patients were interviewed at the health care facilities involved, in rooms reserved specifically for that purpose, by researchers trained in data collection via an instrument with pre-coded closed questions. The instrument was designed to assess the following variables: sociodemographic data (gender, age, and place of residence); willingness to undergo HIV testing; time to diagnosis (hereafter time to diagnosis); history of hemoptysis; history of tuberculosis (for this variable, the self-reported answers-"yes", "no", or "don't know"-were verified against the patient records available at the primary health care clinics or in other patient record systems); use of antituberculosis drugs; cough with expectoration for more than 3 weeks; previous chest X-ray; previous sputum testing; previous use of antituberculosis drugs; and case type (new case, re-treatment after cure, re-treatment after default from treatment, chronic treatment failure, or unknown). All patients were informed that HIV testing is a routine assessment procedure and were invited to undergo said testing. Researchers were trained in the provision of pre- and post-HIV test counseling. The samples collected for HIV testing were sent to a laboratory for diagnostic testing with ELISA. Patients were informed of the HIV test results and, when necessary, were offered counseling and directed to the AIDS treatment facility nearest to their place of residence.

Two sputum samples were collected from each patient at the respective health care facilities. Sputum smears were then examined using Ziehl-Neelsen staining. Procedures for smear microscopy preparation, staining and reading were conducted according to international guidelines.^(^ ^14^ ^,^ ^15^ ^)^ Clinical samples were sent to the Rio Grande do Sul State Referral Laboratory for processing. After decontamination, material was inoculated into two tubes containing Löwenstein-Jensen (LJ) medium. Cultures were incubated at 37°C for up to 6 weeks, until colony growth was observed. Cultures were inspected 48 h after inoculation and weekly until day 42 of incubation. Strain morphology and pigmentation were observed, and the date on which colonies appeared was recorded. These procedures were conducted according to the tuberculosis guidelines established by the Brazilian National Ministry of Health.^(^ ^16^ ^)^ We identified strains of M. tuberculosis by growth inhibition test, using p-nitrobenzoic acid at a concentration of 500 µg per 1 mL of LJ medium, as well as niacin and nitrate tests.^(^ ^15^ ^)^

Indirect susceptibility testing was performed on the samples obtained from the participants. Culture growth on day 28 of incubation determined the final results, which were interpreted in relation to the resistance criteria recommended in the WHO guidelines (i.e., 1%).^(^ ^14^ ^)^ For each lot of LJ medium and each antituberculosis drug tested, DST was also conducted on the reference strain of *M. tuberculosis* (H37Rv), which was thus used as a susceptible control. All laboratories involved in testing used a double-blind method for internal quality control. In addition, 100% of the samples identified as drug-resistant were retested by another referral laboratory, as were 15% of those identified as susceptible.

A database was created using the EpiData^(r)^ program (EpiData Association, Odense, Denmark). Data analyses comprised prevalence estimates, confidence intervals (considered significant at 5%) and group comparisons. Chi-square tests were used in comparisons between individuals infected with resistant strains and those infected with susceptible strains. Measures of association, such as prevalence ratios, were calculated using STATA software, version 10.

The present study was approved by the Research Ethics Committee of the Porto Alegre Municipal Health Department (Protocol no. 001.053413.05.9; approved 16 December, 2005). The nationwide project (the Second National Survey on Antituberculosis Drug Resistance) was approved by the National Committee for Research Ethics (Protocol no. 25000.178623/2004-80; approved 24 May, 2005). All participating patients (or their legal guardians) gave written informed consent.

## Results

**Figure 1:**
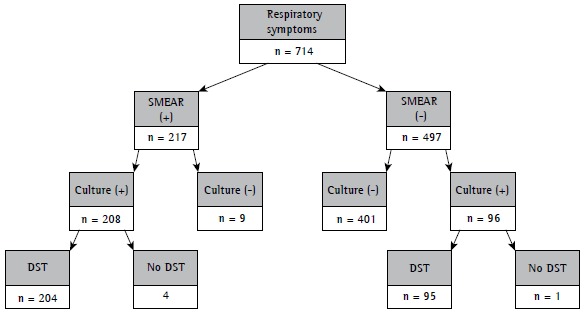
Patient distribution according to laboratory tests conducted in Porto Alegre, 2006-2007. DST: drug susceptibility testing

Of all patients with suspected pulmonary tuberculosis seen at the participating health care facilities, 714 were eligible for participation in the present study. Of those 714 patients, 208 (29.1%) and 96 (13.4%) were found to be smear positive-culture positive and smear negative-culture positive, respectively, 299 (41.9%) subsequently undergoing DST (Figure 1).

Table 1 displays the demographic and clinical characteristics of the survey participants. The majority of participants were young adults, and the male-to-female ratio was 3:1. There were no gender or age differences between the patients with a history of treatment for tuberculosis and those without.

**Table 1:** Clinical and demographic characteristics of participants in the Second National Survey on Antituberculosis Drug Resistance, in Porto Alegre, Brazil. 2006-2007.

Variable	History of tuberculosis treatment		Total
	No	Yes	
	(n = 224^a^)	(n = 75^a^)	(n = 299^a^)
Age (years), mean	35.0	38.0	36.0
Gender, n (%)			
Male	165 (73.6)	56 (74.7)	221 (73.9)
Female	59 (26.4)	19 (25.3)	78 (26.1)
HIV testing in the past 2 months, n (%)			
Yes	37 (18.7)	17 (27.9)	54 (20.8)
No	161 (81.3)	44 (72.1)	205 (79.2)
Consented to HIV testing, n (%)	123 (63.4)	34 (57.6)	157 (62.1)
Time to diagnosis (days), mean	86.6	184.8	110.9
Self-reported history of tuberculosis, n (%)			
Yes	5 (2.4)	72 (100.0)	77 (27.3)
No	202 (97.6)		202 (72.7)
Productive cough for > 3 weeks, n (%)			
Yes	93 (45.4)	67 (91.8)	160 (57.5)
No	112 (54.6)	6 (8.2)	118 (42.5)
Hemoptysis, chest pain, or other symptom of lung disease, n (%)			
Yes	70 (34.1)	55 (73.3)	125 (45.0)
No	135 (65.9)	18 (26.7)	153 (55.0)
Chest X-ray, n (%)			
Yes	125 (61.0)	70 (95.9)	195 (70.1)
No	80 (39.0)	3 (4.1)	83 (29.9)
Previous sputum testing, n (%)			
Yes	70 (34.8)	73 (98.6)	143 (52.0)
No	131 (65.2)	1 (1.4)	132 (48.0)
Antituberculosis medication for > 1 month, n (%)			
Yes	6 (3.1)	73 (98.6)	79 (29.6)
No	187 (96.9)	1 (1.4)	188 (70.4)

a The maximum possible numbers of patients

One fifth of patients reported having undergone prior HIV testing. The frequency of HIV testing in the two months prior to the survey was higher in patients with a history of tuberculosis treatment than in those without. Although the mean time to diagnosis was longer in the patients with a history of tuberculosis than in those without, it was greater than three months in both groups.

Resistance to at least one antituberculosis drug (DR-TB) and combined resistance to at least isoniazid and rifampin (MDR-TB) were observed in 14.0% and 4.7% of the patients, respectively. Drug resistance was eight times greater in the patients with a history of tuberculosis (p = 0.01). Isoniazid monoresistance was more common than was rifampin monoresistance. The prevalence rates of primary and acquired MDR-TB were 2.2% and 12.0%, respectively. Positive HIV test results were seen in 26% of the patients, and the frequency of such results was higher in the patients with a history of tuberculosis (Table 2). As can be seen in Table 3, HIV infection was not found to be associated with DR-TB or MDR-TB. However, the time to diagnosis was associated with DR-TB and MDR-TB. In patients with a history of hemoptysis, there was a higher prevalence of DR-TB but not of MDR-TB.

**Table 2:** Prevalence of combined, primary, and acquired resistance to antituberculosis drugs and HIV infection among participants in the Second National Survey on Antituberculosis Drug Resistance, in Porto Alegre, Brazil. 2006-2007.

Variable	No history of TB treatment			History of TB treatment			Combined resistance		
	(primary resistance)			(acquired resistance)					
	n	Prevalence, %	95% CI	n	Prevalence, %	95% CI	n	Prevalence, %	95% CI
Drug susceptibility	224	91.5	87.9-95.2	75	68.0	57.2-78.8	299	85.6	81.7-89.7
Any resistance	224	8.5	4.8-12.1	75	32.0	21.2- 42.8	299	14.4	10.4-18.4
INH	224	7.1	3.7-10.5	75	29.3	18.8-39.9	299	12.7	8.9-16.5
RIF	224	2.2	0.3-4.2	75	13.3	5.4-21.2	299	5.0	2.5-7.5
Monoresistance	224	4.9	2.0-7.8	75	18.7	9.6-27.7	299	8.4	5.2-11.5
INH	224	4.9	2.0-7.8	75	17.3	8.6-26.1	299	8.0	4.9-11.1
RIF	224	0.0	0.0-0.0	75	1.3	0.0-3.9	299	0.3	0.0-0.9
Multidrug resistance									
INH+RIF	224	2.2	0.3-4.2	75	12.0	4.5-19.5	299	4.7	2.3-7.1
Resistance to 1 drug	224	4.9	2.0-7.8	75	18.7	9.6-27.7	299	8.4	5.2-11.5
Resistance to 2 drugs	224	2.2	0.3-4.2	75	12.0	4.5-9.5	299	4.7	2.3-7.1
HIV infection	185	23.8	17.6-30.0	67	32.8	23.1-44.4	252	26.2	20.7-31.6

INH : isoniazid

RIF : rifampin

**Table 3:** Variables associated with drug resistance and multidrug resistance, in bivariate and multivariate analyses, among participants in the Second National Survey on Antituberculosis Drug Resistance, in Porto Alegre, Brazil. 2006-2007.

Variable	n	Bivariate analysis				Multivariate analysis			
		Resistance		Multidrug resistance		Resistance		Multidrug resistance	
		PR	95% CI	PR	95% CI	PR	95% CI	PR	95% CI
Re-treatment									
No	224	1.00		1.00		1.00			
Yes	75	5.08	2.58-9.98	5.97	1.93-18.44	4.10	1.61-10.41	4.96	0.87-28.44
HIV infection									
No	186	1.00		1.00		1.00		1.00	
Yes	66	0.72	0.31-1.65	1.22	0.30-4.85	0.31	0.09-1.08	0.20	0.01-2.63
Time to diagnosis (days)	258	1.00^a^	1.00-1.00^b^	1.00^c^	1.00-1.00^d^	1.00^e^	1.00-1.00^f^	1.00^g^	1.00-1.00^h^
History of hemoptysis									
No	153	1.00		1.00		1.00		1.00	
Yes	125	2.03	1.03-4.03	2.30	0.75-7.04	0.94	0.37-2.37	0.50	0.09-2.62

PR : prevalence ratio

**aobserved value:** : 1.001

**bobserved value:** : 1.0003-1.002

**cobserved value:** : 1.001

**dobserved value:** : 1.0004-1.003

**eobserved value:** : 1.001

**fobserved value:** : 1.00002-1.002

**gobserved value:** : 1.001

**hobserved value:** : 1.0001-1.003

In summary, the bivariate analyses indicated that the following variables were associated with DR-TB: tuberculosis re-treatment; time to diagnosis; and history of hemoptysis. We also found that re-treatment was associated with MDR-TB, as was the time to diagnosis. Multivariate analyses revealed that DR-TB was independently associated with tuberculosis re-treatment and with the time to diagnosis. When this calculation was adjusted for the influence of other variables (Table 3), only the time to diagnosis was associated with MDR-TB.

## Discussion

The prevalence rates of primary and acquired MDR-TB observed in the present study (2.2% and 12.0%, respectively) were higher than those reported in the First National Survey on Antituberculosis Drug Resistance (1.1% and 7.9%, respectively), which was carried out in Brazil in 1996, and in the International WHO-IUATLD report, which was conducted in 58 countries between 1994 and 1999 (1.0% and 9.3%, respectively). ^(^ ^3^ ^)^ However, the present estimates of primary and acquired MDR-TB prevalence were lower than the respective rates of 2.9% and 15.3% reported in the WHO-IUATLD survey conducted between 2002 and 2007.^(^ ^3^ ^)^ The prevalence rates of MDR-TB in Lithuania and Azerbaijan, for instance, were 14.4% and 22.3%, respectively.^(^ ^3^ ^,^ ^10^ ^)^ The high prevalence of primary MDR-TB found in the present study (2.2%) might be attributable to the increase in the rate of default from treatment observed over the last 10 years in the city of Porto Alegre.^(^ ^8^ ^)^

Our results suggest that DR-TB is associated with re-treatment and with a longer time to diagnosis. These conditions, in turn, might represent the consequences of delayed diagnosis and lack of prompt treatment in cases of tuberculosis, as has been suggested in previous studies.^(^ ^17^ ^-^ ^19^ ^)^ Although difficulties in the diagnosis of tuberculosis and the detection of resistance to antituberculosis drugs-even after the implementation of the directly observed treatment, short-course (DOTS) strategy or the DOTS-plus strategy-have been reported in a number of countries, few studies have evaluated the variables associated with delayed detection of DR-TB.^(^ ^20^ ^)^ A recent analysis of tuberculosis transmission and delayed diagnosis suggested that the duration of this delay is the main obstacle in controlling the tuberculosis epidemic.^(^ ^17^ ^)^ Storla et al.^(^ ^19^ ^)^ also suggested that repeated attempts by patients to seek treatment at the same level of health care and the inconclusive test results obtained at that level are responsible for delaying the diagnosis of tuberculosis.

In the present sample, the mean time from symptom onset to a diagnosis of tuberculosis was 110.9 days, which is longer than the delays reported for other developing countries (61.3 days) and for developed countries (67.8 days).^(^ ^21^ ^)^ This figure is also higher than (or comparable to) that reported in surveys conducted in other major Brazilian cities: 68 days in Rio de Janeiro; 110 days in Vitória; and 90 days in Recife.^(^ ^7^ ^,^ ^22^ ^,^ ^23^ ^)^

Among our sample of patients in the city of Porto Alegre, the association found between a history of tuberculosis treatment and the time to diagnosis, which was 184.8 days for those with such a history, has not been observed in surveys conducted in other Brazilian cities, such as Rio de Janeiro.^(^ ^23^ ^)^ One of the risk factors for delayed detection of DR-TB is an increased probability of transmission to individuals at home or in hospital environments, or even in prisons or shelters. The chain of transmission continues and leads to further contamination and aggravation of existing cases of tuberculosis, contributing to the worldwide epidemic. In Porto Alegre, delayed detection of DR-TB is one of the main aggravating factors of the epidemiological situation. This might be attributable to flaws in the health care system, because patients often continue to visit health care facilities until receiving a diagnosis. Therefore, variables related to patient behavior and to the health care system contribute to delays in the detection of DR-TB.

The mean age and the male-to-female ratio observed in the present study were similar to those described by the Porto Alegre Municipal Health Department, as well as in the national and international literature.^(^ ^20^ ^,^ ^24^ ^-^ ^26^ ^)^ A high number of HIV-infected patients were also found in the sample. That might be explained by the type of health care facilities investigated in the current study. It is possible that some of those facilities had multidisciplinary teams and treated patients who were referred from other health care facilities. We also found that patients with a history of tuberculosis treatment were more likely to have undergone HIV testing, probably because they sought diagnostic and treatment services via tuberculosis control programs within which HIV testing has become a routine requirement.

The responses to the screening questions for a history of tuberculosis treatment indicated that 61% of previously untreated patients had previously undergone chest X-ray, even though the Brazilian National Ministry of Health does not recommend X-ray screening in patients with a productive cough and suspected tuberculosis. ^(^ ^13^ ^)^ In the present study, a history of tuberculosis symptoms was investigated through questions related to hemoptysis, chest pain, and other symptoms of pulmonary tuberculosis. Such symptoms were identified in 45% of the study sample and were more common in patients with a history of tuberculosis treatment, as would be expected. It is of note that we also investigated hemoptysis, which is a less common symptom that presents later in the course of illness.^(^ ^27^ ^)^

The frequency of HIV infection among our study subjects was elevated but lower than that reported in the Brazilian National Case Registry Database for Porto Alegre.^(^ ^8^ ^)^ Our results differed from those in the literature in that the incidence of DR-TB in HIV-infected patients with a history of tuberculosis treatment was higher in our sample (32.8%). A study conducted in the state of Santa Catarina (also in southern Brazil) found that the prevalence of HIV infection was higher in patients who had never been treated for tuberculosis than in those with a history of tuberculosis treatment (20% vs. 9%).^(^ ^28^ ^)^ Our findings also support the hypothesis that the frequency of DR-TB is higher in regions where there are high rates of default from treatment.

The results of the present study call for awareness of tuberculosis control strategies by health care authorities, managers, and workers, in order to improve the health situation in the region studied. There is an urgent need to increase treatment coverage, reduce the rate of default from treatment, and identify strategies for early diagnosis of DR-TB and MDR-TB at primary health care clinics and hospitals in Porto Alegre. Effective strategies could include new diagnostic tests (liquid culture or molecular testing) or the use of clinical prediction rules. The latter method was suggested by researchers in Peru, a country with a high prevalence of DR-TB, where significant technical and political efforts have been made toward the implementation of programs for the control of DR-TB and MDR-TB.^(^ ^29^ ^)^

In the present study, it was possible to analyze the epidemiological behavior of DR-TB and the variables associated with this condition in a group of patients included in the Second National Survey on Antituberculosis Drug Resistance, which was conducted in the city of Porto Alegre. A longer time from symptom onset to a diagnosis of tuberculosis and history of tuberculosis treatment were found to be associated with the occurrence of DR-TB and MDR-TB. If our results are corroborated by other studies conducted in Brazil, these variables could be used as predictors of MDR-TB, thus contributing to the investigation and implementation of appropriate drug therapy. In addition, these findings could promote lower morbidity and mortality rates, as well as lowering the risk of tuberculosis transmission within the community.
