# Supplementary material for: Drug-resistant tuberculosis in subjects included in the Second National Survey on Antituberculosis Drug Resistance in Porto Alegre, Brazil
Source: J Bras Pneumol. 2014 Mar-Apr;40(2):155–63. doi: 10.1590/S1806-37132014000200009 (PMC4083645; doi:10.1590/S1806-37132014000200009)
Supplement: Translation

# Translation: Tuberculose resistente em pacientes incluídos no II Inquérito Nacional de Resistência aos Fármacos Antituberculose realizado em Porto Alegre, Brasil*

**Authors:** Vania Celina Dezoti Micheletti, José da Silva Moreira, Marta Osório Ribeiro, Afranio Lineu Kritski, José Ueleres Braga

**Affiliations:** Universidade Federal do Rio Grande do Sul, Porto Alegre (RS) Brasil; Departamento de Pneumologia. Universidade Federal do Rio Grande do Sul, Porto Alegre (RS) Brasil; Instituto de Microbiologia, Universidade Federal do Rio de Janeiro, Rio de Janeiro (RJ) Brasil; Departamento de Pneumologia, Faculdade de Medicina, Universidade Federal do Rio de Janeiro, Rio de Janeiro (RJ) Brasil; Departamento de Saúde Coletiva, Universidade do Estado do Rio de Janeiro; e Pesquisador em Saúde Pública. Fundação Oswaldo Cruz, Rio de Janeiro (RJ) Brasil

**Keywords:** Tuberculose/diagnóstico, Resistência a medicamentos, HIV

## Abstract

**OBJETIVO:**

Descrever a prevalência de tuberculose multirresistente (TBMR) em pacientes com tuberculose em uma importante cidade brasileira através do II Inquérito Nacional de Resistência aos Fármacos Antituberculose, assim como as características sociais, demográficas e clínicas desses pacientes.

**MÉTODOS:**

De 2006 a 2007, amostras clínicas de pacientes de três hospitais e das cinco unidades básicas de saúde participantes do inquérito realizado em Porto Alegre foram coletadas e submetidas ao teste de sensibilidade aos fármacos. A confirmação das espécies de micobactérias ocorreu por métodos bioquímicos.

**RESULTADOS:**

Foram incluídos 299 pacientes. Desses, 221 (73,9%) eram homens e 77 (27,3%) tinham história de tuberculose. A idade média foi de 36 anos. Dos 252 pacientes testados para HIV, 66 (26,2%) estavam infectados. A prevalência da TBMR na amostra geral foi de 4,7% (IC95%: 2,3-7,1); enquanto essa foi de 2,2% (IC95%: 0,3-4,2) nos pacientes virgens de tratamento e de 12,0% (IC 95%: 4,5-19,5) naqueles com história de tratamento antituberculose. A análise multivariada mostrou que história de tuberculose e maior tempo para o diagnóstico associaram-se a TBMR.

**CONCLUSÕES:**

Caso esses resultados sejam confirmados em outros estudos no Brasil, a história de tratamento antituberculose e o maior tempo para o diagnóstico poderão ser utilizados como preditores de TBMR.

## Introdução

Houve um recrudescimento da tuberculose no final dos anos 1980, o que levou a Organização Mundial da Saúde (OMS) a declarar a mesma um problema emergencial de saúde pública em 1993. ^(^ ^1^ ^)^ No início de 1994, a OMS também iniciou o Projeto Global de Vigilância à Resistência aos Fármacos Antituberculose, em colaboração com a *International Union against Tuberculosis and Lung Disease* (IUATLD).^(^ ^2^ ^)^ Entre 1994 e 1999, a OMS e a IUATLD compilaram dados sobre resistência provenientes de inquéritos realizados em 58 países.^(^ ^3^ ^)^ Constatou-se que a prevalência média de tuberculose multirresistente primária (TBMR) em pacientes sem história de tratamento antituberculose foi de 1,0% (variação, 0-14,1%), e que a prevalência média de TBMR adquirida foi de 9,3% (variação, 0-48,2%).^(^ ^3^ ^)^

Estudos realizados entre 2002 e 2006, os quais envolveram 90.000 pacientes em 81 países, demonstraram um aumento da prevalência estimada de tuberculose resistente (DR-TB, do inglês *drug-resistant tuberculosis*).^(^ ^4^ ^,^ ^5^ ^)^ Em 2005, ocorreram 500.000 casos novos de TBMR no mundo, correspondendo a 5% do número total de casos de tuberculose. No mesmo ano, a prevalência de TBMR primária foi de 2,9% (variação, 2,2-3,6%), enquanto a de TBMR adquirida foi de 15,3% (variação, 9,6-21,1%), respectivamente.^(^ ^4^ ^)^

Em 2006, casos de tuberculose extensivamente resistente (XDR-TB, do inglês *extensively drug-resistant tuberculosis*) foram relatados na África do Sul, principalmente em pacientes hospitalizados infectados pelo HIV. Até 2009, casos de XDR-TB haviam sido relatados em várias outras regiões do mundo.^(^ ^6^ ^)^ As pesquisas também mostraram que as taxas de óbito foram maiores em países com prevalência elevada de coinfecção tuberculose/HIV (que incluía casos de TBMR ou XDR-TB em indivíduos infectados pelo HIV), enfatizando a necessidade de intervenções eficazes para a prevenção e o tratamento da infecção por cepas de *Mycobacterium tuberculosis* resistentes.^(^ ^7^ ^)^

No Brasil, reduções das taxas de incidência e mortalidade sugerem que a situação da tuberculose melhorou nos últimos dez anos. Porém, em certas regiões metropolitanas do país, não houve nenhuma melhora.^(^ ^2^ ^)^ Por exemplo, na cidade de Porto Alegre, no sul do Brasil, a incidência de tuberculose aumentou de 97/100.000 habitantes para 116/100.000 habitantes entre 2001 e 2009, sendo que 30% de todos os casos de tuberculose notificados para a cidade foram diagnosticados em hospitais. Esse aumento foi acompanhado por uma alta prevalência de coinfecção tuberculose/HIV, uma diminuição da taxa de cura da tuberculose (de 69% para 65% de todos os casos tratados) e um aumento da taxa de abandono do tratamento (de 15% para 20%).^(^ ^8^ ^)^

Em 1996, foi realizado no Brasil o I Inquérito Nacional de Resistência aos Fármacos Antituberculose.^(^ ^9^ ^)^ Os participantes foram recrutados em 13 unidades de saúde de todo o país, e aproximadamente 6.000 cepas de *M. tuberculosis* foram identificadas.^(^ ^9^ ^)^ As taxas de prevalência de TBMR primária e adquirida foram de 1.1% e 7.9%, respectivamente.^(^ ^10^ ^)^ Porém, o inquérito não avaliou a prevalência de infecção pelo HIV e limitou-se a pacientes atendidos em unidades básicas de saúde.^(^ ^10^ ^)^ No Sul do Brasil, as taxas de prevalência de TBMR primária e adquirida (0,8% e 5,8%, respectivamente) foram menores do que a prevalência nacional. ^(^ ^10^ ^)^ Desde então, não foi realizado nenhum outro estudo epidemiológico (de base populacional) sobre resistência aos fármacos antituberculose em nenhuma das grandes cidades do Sul do Brasil. Portanto, o presente estudo teve como objetivo caracterizar a prevalência de DR-TB e de TBMR em Porto Alegre, onde a eficácia dos programas de controle da tuberculose diminuiu significativamente nos últimos anos. O presente estudo também teve como objetivo identificar a prevalência de infecção pelo HIV e quaisquer características demográficas ou clínicas associadas à resistência aos fármacos antituberculose em uma população recrutada em unidades básicas de saúde e em hospitais.

## Métodos

Os dados analisados no presente estudo foram coletados em Porto Alegre como parte do II Inquérito Nacional de Resistência aos Fármacos Antituberculose, realizado entre 2006 e 2007. Entre março de 2006 e dezembro de 2007, pacientes foram recrutados em cinco unidades básicas de saúde e em três hospitais públicos. Todos os pacientes forneceram amostras de escarro para baciloscopia e para cultura de micobactérias. As amostras também foram testadas para resistência a rifampicina, estreptomicina, etambutol e isoniazida. Porém, em razão da baixa reprodutibilidade dos testes de resistência à estreptomicina e ao etambutol, os resultados dos mesmos não foram considerados no presente estudo.?Com base nos resultados do exame bacteriológico, definimos DR-TB como resistência a qualquer fármaco antituberculose e TBMR como resistência a (pelo menos) isoniazida e rifampicina. A presença de organismos resistentes a um ou mais fármacos em pacientes sem história de tratamento antituberculose, ou com tratamentos anteriores com duração de um mês ou menos, foi classificada como resistência primária. A presença de microrganismos resistentes em pacientes com história de tratamentos antituberculose com duração de mais de um mês foi classificada como resistência adquirida.

Dadas as diferenças na prevalência esperada de resistência à rifampicina em pacientes novos (resistência primária) e em pacientes em retratamento (resistência adquirida), tamanhos amostrais mínimos foram calculados para esses dois grupos. Esses cálculos foram realizados por meio de um método de amostragem por conglomerados proporcional ao tamanho da população, levando-se em consideração o tamanho das instalações diagnósticas para tuberculose e, consequentemente, o número de pacientes internados para diagnóstico e tratamento em cada serviço de saúde.^(^ ^11^ ^,^ ^12^ ^)^

Os participantes foram recrutados em cinco unidades básicas de saúde (Modelo, Navegantes, Instituto de Proteção e Assistência à Infância, Vila dos Comerciários e Sanatório), bem como em três hospitais (o Hospital Nossa Senhora da Conceição de Porto Alegre, o Hospital Sanatório Partenon e o Hospital de Clínicas de Porto Alegre da Faculdade de Medicina da Universidade Federal do Rio Grande do Sul), todos localizados em Porto Alegre. Todos os pacientes que procuraram algum desses serviços de saúde durante o período de recrutamento e apresentaram suspeita de tuberculose pulmonar foram considerados elegíveis para participar. Definiu-se suspeita de tuberculose pulmonar como a presença de sintomas respiratórios ou de sinais clínicos ou radiológicos de tuberculose, de acordo com as Diretrizes Brasileiras para o Controle da Tuberculose.^(^ ^13^ ^)^ Foram realizadas culturas de micobactérias para todas as amostras clínicas, independentemente dos resultados da baciloscopia de escarro.

Os pacientes considerados elegíveis foram incluídos no estudo caso preenchessem um dos dois seguintes critérios: ser classificado como virgem de tratamento (sem historia de tratamento antituberculose) com tuberculose pulmonar cultura positiva (independentemente dos resultados da baciloscopia); e ter história de tratamento antituberculose (recidiva ou história de abandono de tratamento antituberculose), apresentar tuberculose pulmonar cultura positiva ou ter usado fármacos antituberculose nos 30 dias anteriores à participação no inquérito e à coleta de escarro. Os critérios de exclusão aplicados foram os seguintes: ter idade inferior a 18 anos; estar grávida; e ter cultura negativa (independentemente dos resultados da baciloscopia de escarro) ou não ter resultado de teste de sensibilidade aos fármacos (TS) - ou seja, TS não realizado segundo o Manual de Recomendações para o Controle da Tuberculose no Brasil.^(^ ^13^ ^)^ As amostras de escarro foram coletadas antes do início do tratamento. Não foi necessário que os pacientes consentissem a realização do teste de HIV para participarem do estudo.

Os pacientes foram entrevistados nos serviços de saúde envolvidos, em salas reservadas especificamente para esse fim, por pesquisadores treinados na coleta de dados por meio de um instrumento com questões fechadas e pré-codificadas. O instrumento foi elaborado para avaliar as seguintes variáveis: dados sociodemográficos (sexo, idade e local de residência); disposição em realizar o teste de HIV; tempo para o diagnóstico; história de hemoptise; história de tuberculose (para essa variável, as respostas de autorrelato - "sim", "não" ou "não sei" - foram confrontadas com os registros de pacientes disponíveis nas unidades básicas de saúde ou em outros sistemas de registros de pacientes); uso de fármacos antituberculose; tosse com expectoração por mais do que 3 semanas; radiografia de tórax prévia; exame de escarro prévio; uso prévio de fármacos antituberculose; e tipo de caso (caso novo, retratamento após cura, retratamento após abandono do tratamento, falha crônica do tratamento ou desconhecido). Todos os pacientes foram informados que o teste de HIV é um procedimento de avaliação de rotina e foram convidados a realizar o dito teste. Os pesquisadores foram treinados na prestação de aconselhamento pré e pós-teste de HIV. As amostras coletadas para o teste de HIV foram enviadas a um laboratório para teste diagnóstico pelo método ELISA. Os pacientes foram informados dos resultados do teste de HIV, e, quando necessário, foi-lhes oferecido aconselhamento e eles foram direcionados à unidade de tratamento da AIDS mais próxima ao seu local de residência.

Duas amostras de escarro foram coletadas de cada paciente nos respectivos serviços de saúde. Os esfregaços de escarro foram então examinados por coloração de Ziehl-Neelsen. Os procedimentos de preparação, coloração e leitura das baciloscopias foram realizados de acordo com diretrizes internacionais.^(^ ^14^ ^,^ ^15^ ^)^ As amostras clínicas foram enviadas ao Laboratório de Referência do Estado do Rio Grande do Sul para processamento. Após a descontaminação, o material foi inoculado em dois tubos contendo meio Löwenstein-Jensen (LJ). As culturas foram incubadas a 37°C por até 6 semanas, até que se observou o crescimento de colônias. As culturas foram inspecionadas 48 h após a inoculação e semanalmente até o 42º dia de incubação. Foram observadas a morfologia e a pigmentação das cepas, e registrou-se a data de surgimento das colônias. Esses procedimentos foram realizados de acordo com as diretrizes para tuberculose estabelecidas pelo Ministério da Saúde do Brasil.^(^ ^16^ ^)^ As cepas de *M. tuberculosis* foram identificadas pelo teste de inibição de crescimento, utilizando-se ácido nitrobenzoico na concentração de 500 µg para 1 mL de meio LJ, bem como por provas de niacina e nitrato.^(^ ^15^ ^)^

O teste de sensibilidade indireto foi realizado nas amostras obtidas dos participantes. O crescimento em cultura no 28º dia de incubação determinou os resultados finais, que foram interpretados em relação aos critérios de resistência recomendados nas diretrizes da OMS (ou seja, 1%).^(^ ^14^ ^)^ Para cada lote de meio LJ e cada fármaco antituberculose testado, também se realizou TS na cepa de referência de *M. tuberculosis* (H37Rv), que foi então utilizada como controle sensível. Todos os laboratórios envolvidos nos testes utilizaram um método duplo-cego para controle interno de qualidade. Além disso, 100% das amostras identificadas como resistentes foram retestadas por outro laboratório de referência, bem como o foram 15% daquelas identificadas como sensíveis.

Foi criado um banco de dados com o programa EpiData^(r)^ (EpiData Association, Odense, Dinamarca). As análises de dados incluíram estimativas de prevalência, intervalos de confiança (considerados significativos a 5%) e comparações entre grupos. Os testes do qui-quadrado foram usados para as comparações entre indivíduos com cepas resistentes e aqueles infectados por cepas sensíveis. As medidas de associação, tais como razões de prevalência, foram calculadas com o software STATA, versão 10.

O presente estudo foi aprovado pelo Comitê de Ética em Pesquisa da Secretaria Municipal de Saúde de Porto Alegre (protocolo nº 001.053413.05.9; aprovado em 16 de dezembro de 2005). O projeto nacional (o II Inquérito Nacional de Resistência aos Fármacos Antituberculose) foi aprovado pelo Comitê Nacional de Ética em Pesquisa (protocolo nº 25000.178623/2004-80; aprovado em 24 de maio de 2005). Todos os pacientes que participaram do estudo (ou seus responsáveis) assinaram um termo de consentimento livre e esclarecido.

## Resultados

De todos os pacientes com suspeita de tuberculose pulmonar atendidos nos serviços de saúde participantes, 714 foram considerados elegíveis para participar do presente estudo. Desses 714 pacientes, 208 (29,1%) e 96 (13,4%) apresentaram baciloscopia positiva-cultura positiva e baciloscopia negativa-cultura positiva, respectivamente, sendo que 299 (41,9%) posteriormente foram submetidos ao TS (Figura 1).

**Figura 1:** Distribuição dos pacientes de acordo com os testes laboratoriais realizados em Porto Alegre, 2006-2007. TS: teste de sensibilidade aos fármacos

A Tabela 1 mostra as características demográficas e clínicas dos participantes do inquérito. A maioria dos participantes era de jovens adultos, e a razão entre os sexos masculino e feminino foi de 3:1. Não houve diferenças em relação ao sexo ou idade entre os pacientes com e sem história de tratamento antituberculose.

**Tabela 1:** Características clínicas e demográficas dos participantes do II Inquérito Nacional de Resistência aos Fármacos Antituberculose, realizado em Porto Alegre, Brasil. 2006-2007.

Variável	História de tratamento antituberculose		Total
	Não	Sim	
	(n = 224^a^)	(n = 75^a^)	(n = 299^a^)
Idade (anos), média	35,0	38,0	36,0
Sexo, n (%)			
Masculino	165 (73,6)	56 (74,7)	221 (73,9)
Feminino	59 (26,4)	19 (25,3)	78 (26,1)
Teste de HIV nos últimos 2 meses, n (%)			
Sim	37 (18,7)	17 (27,9)	54 (20,8)
Não	161 (81,3)	44 (72,1)	205 (79,2)
Consentiu a realização do teste de HIV, n (%)	123 (63,4)	34 (57,6)	157 (62,1)
Tempo para o diagnóstico (dias), média	86,6	184,8	110,9
História autorrelatada de tuberculose, n (%)			
Sim	5 (2,4)	72 (100,0)	77 (27,3)
Não	202 (97,6)		202 (72,7)
Tosse produtiva por > 3 semanas, n (%)			
Sim	93 (45,4)	67 (91,8)	160 (57,5)
Não	112 (54,6)	6 (8,2)	118 (42,5)
Hemoptise, dor torácica ou outro sintoma de doença pulmonar, n (%)			
Sim	70 (34,1)	55 (73,3)	125 (45,0)
Não	135 (65,9)	18 (26,7)	153 (55,0)
Radiografia de tórax, n (%)			
Sim	125 (61,0)	70 (95,9)	195 (70,1)
Não	80 (39,0)	3 (4,1)	83 (29,9)
Exame de escarro prévio, n (%)			
Sim	70 (34,8)	73 (98,6)	143 (52,0)
Não	131 (65,2)	1 (1,4)	132 (48,0)
Medicação antituberculose por > 1 mês, n (%)			
Sim	6 (3,1)	73 (98,6)	79 (29,6)
Não	187 (96,9)	1 (1,4)	188 (70,4)

a Números máximos possíveis de pacientes; em razão de dados incompletos, os valores para algumas variáveis são baseados em números menores.

Um quinto dos pacientes relatou ter sido testado para HIV anteriormente. A frequência de teste de HIV nos dois meses anteriores ao inquérito foi maior nos pacientes com do que naqueles sem história de tratamento antituberculose. Embora o tempo médio para o diagnóstico tenha sido maior nos pacientes com do que naqueles sem história de tratamento antituberculose, ele foi maior do que três meses em ambos os grupos.

Resistência a pelo menos um fármaco antituberculose (DR-TB) e resistência combinada a pelo menos isoniazida e rifampicina (TBMR) foram observadas em 14,0% e 4,7% dos pacientes, respectivamente. A resistência foi oito vezes maior nos pacientes com história de tuberculose (p = 0,01). Monorresistência à isoniazida foi mais frequente do que monorresistência à rifampicina. As taxas de prevalência de TBMR primária e adquirida foram de 2,2% e 12,0%, respectivamente. Resultados positivos no teste de HIV foram observados em 26% dos pacientes, e a frequência desses resultados foi maior nos pacientes com história de tuberculose (Tabela 2). Como se pode observar na Tabela 3, a infecção pelo HIV não mostrou associação com DR-TB ou com TBMR. Porém, o tempo para o diagnóstico associou-se a DR-TB e a TBMR. Em pacientes com história de hemoptise, houve maior prevalência de DR-TB mas não de TBMR.

**Tabela 2:** Prevalência de resistência combinada, primária e adquirida a fármacos antituberculose e de infecção pelo HIV entre os participantes do II Inquérito Nacional de Resistência aos Fármacos Antituberculose, realizado em Porto Alegre, Brasil. 2006-2007.

Variável	Sem história de tratamento anti-TB			História de tratamento anti-TB			Resistência combinada		
	(resistência primária)			(resistência adquirida)					
	n	Prevalência, %	IC95%	n	Prevalência, %	IC95%	n	Prevalência, %	IC95%
Sensibilidade aos fármacos	224	91,5	87,9-95,2	75	68,0	57,2-78,8	299	85,6	81,7-89,7
Qualquer resistência	224	8,5	4,8-12,1	75	32,0	21,2- 42,8	299	14,4	10,4-18,4
INH	224	7,1	3,7-10,5	75	29,3	18,8-39,9	299	12,7	8,9-16,5
RIF	224	2,2	0,3-4,2	75	13,3	5,4-21,2	299	5,0	2,5-7,5
Monorresistência	224	4,9	2,0-7,8	75	18,7	9,6-27,7	299	8,4	5,2-11,5
INH	224	4,9	2,0-7,8	75	17,3	8,6-26,1	299	8,0	4,9-11,1
RIF	224	0,0	0,0-0,0	75	1,3	0,0-3,9	299	0,3	0,0-0,9
Multirresistência									
INH+RIF	224	2,2	0,3-4,2	75	12,0	4,5-19,5	299	4,7	2,3-7,1
Resistência a 1 fármaco	224	4,9	2,0-7,8	75	18,7	9,6-27,7	299	8,4	5,2-11,5
Resistência a 2 fármacos	224	2,2	0,3-4,2	75	12,0	4,5-9,5	299	4,7	2,3-7,1
Infecção pelo HIV	185	23,8	17,6-30,0	67	32,8	23,1-44,4	252	26,2	20,7-31,6

INH : isoniazida

RIF : rifampicina

**Tabela 3:** Variáveis associadas à resistência e a multirresistência, nas análises bivariada e multivariada, entre os participantes do II Inquérito Nacional de Resistência aos Fármacos Antituberculose, realizado em Porto Alegre, Brasil. 2006-2007.

Variável	n	Análise bivariada				Análise multivariada			
		Resistência		Multirresistência		Resistência		Multirresistência	
		RP	IC95%	RP	IC95%	RP	IC95%	RP	IC95%
Retratamento									
Não	224	1,00		1,00		1,00			
Sim	75	5,08	2,58-9,98	5,97	1,93-18,44	4,10	1,61-10,41	4,96	0,87-28,44
Infecção pelo HIV									
Não	186	1,00		1,00		1,00		1,00	
Sim	66	0,72	0,31-1,65	1,22	0,30-4,85	0,31	0,09-1,08	0,20	0,01-2,63
Tempo para o diagnóstico (dias)	258	1,00^a^	1,00-1,00^b^	1,00^c^	1,00-1,00^d^	1,00^e^	1,00-1,00^f^	1,00^g^	1,00-1,00^h^
História de hemoptise									
Não	153	1,00		1,00		1,00		1,00	
Sim	125	2,03	1,03-4,03	2,30	0,75-7,04	0,94	0,37-2,37	0,50	0,09-2,62

RP : razão de prevalência

a valor observado: 1,001

b valor observado: 1,0003-1,002

c valor observado: 1,001

d valor observado: 1,0004-1,003

e valor observado: 1,001

f valor observado: 1,00002-1,002

g valor observado: 1,001

h valor observado: 1,0001-1,003

Em resumo, as análises bivariadas indicaram que as seguintes variáveis associaram-se a DR-TB: retratamento antituberculose, tempo para o diagnóstico e história de hemoptise. Constatamos também que o retratamento associou-se a TBMR, bem como o tempo para o diagnóstico. As análises multivariadas revelaram que a DR-TB associou-se de forma independente ao retratamento antituberculose e ao tempo para o diagnóstico. Quando se ajustou esse cálculo para a influência de outras variáveis (Tabela 3), apenas o tempo para o diagnóstico associou-se a TBMR.

## Discussão

As taxas de prevalência de TBMR primária e adquirida observadas no presente estudo (2,2% e 12,0%, respectivamente) foram maiores do que aquelas relatadas no I Inquérito Nacional de Resistência aos Fármacos Antituberculose (1,1% e 7,9%, respectivamente), realizado no Brasil em 1996, e no relatório internacional da OMS/IUATLD, realizado em 58 países entre 1994 e 1999 (1,0% e 9,3%, respectivamente).^(^ ^3^ ^)^ Porém, as estimativas de prevalência de TBMR primária e adquirida do presente estudo foram menores do que as respectivas taxas de 2,9% e 15,3% relatadas no inquérito da OMS/IUATLD realizado entre 2002 e 2007.^(^ ^3^ ^)^ As taxas de prevalência de TBMR na Lituânia e no Azerbaijão, por exemplo, foram de 14,4% e 22,3%, respectivamente.^(^ ^3^ ^,^ ^10^ ^)^ A alta prevalência de TBMR primária encontrada no presente estudo (2,2%) deve-se possivelmente ao aumento da taxa de abandono do tratamento observado nos últimos 10 anos em Porto Alegre.^(^ ^8^ ^)^

Nossos resultados sugerem que a DR-TB associa-se ao retratamento e ao maior tempo para o diagnóstico. Essas condições, por sua vez, possivelmente representam as consequências do atraso no diagnóstico e da falta de tratamento imediato em casos de tuberculose, como sugerido em estudos prévios.^(^ ^17^ ^-^ ^19^ ^)^ Embora dificuldades no diagnóstico da tuberculose e na detecção de resistência aos fármacos antituberculose - mesmo após a implantação da estratégia *directly observed treatment, short-course* (DOTS, tratamento supervisionado) ou da estratégia DOTS-plus - tenham sido relatadas em muitos países, poucos estudos têm avaliado as variáveis associadas à detecção tardia da DR-TB.^(^ ^20^ ^)^ Uma recente análise sobre transmissão da tuberculose e atraso no diagnóstico sugeriu que a duração desse atraso é o principal obstáculo para o controle da epidemia de tuberculose.^(^ ^17^ ^)^ Storla et al.^(^ ^19^ ^)^ também sugeriram que repetidas tentativas de busca por tratamento no mesmo nível de assistência á saúde feitas pelos pacientes e os resultados inconclusivos dos testes obtidos nesse nível são responsáveis pelo atraso no diagnóstico da tuberculose.

Na presente amostra, o tempo médio entre o início dos sintomas e o diagnóstico de tuberculose foi de 110,9 dias, que é maior do que os atrasos relatados para outros países em desenvolvimento (61,3 dias) e para países desenvolvidos (67,8 dias).^(^ ^21^ ^)^ Esse valor também é maior do que (ou comparável a) aquele relatado em inquéritos realizados em outras importantes cidades brasileiras: 68 dias no Rio de Janeiro, 110 dias em Vitória e 90 dias em Recife.^(^ ^7^ ^,^ ^22^ ^,^ ^23^ ^)^

A associação encontrada em nossa amostra de pacientes em Porto Alegre entre a história de tratamento antituberculose e o tempo para o diagnóstico, que foi de 184,8 dias para aqueles com essa história, não foi observada em inquéritos realizados em outras cidades brasileiras, tais como o Rio de Janeiro.^(^ ^23^ ^)^ Um dos fatores de risco para a detecção tardia da DR-TB é um aumento da probabilidade de transmissão para indivíduos no domicílio e em ambientes hospitalares, ou mesmo em prisões ou abrigos. A cadeia de transmissão continua e leva a uma maior contaminação e agravamento dos casos existentes de tuberculose, contribuindo para a epidemia mundial. Em Porto Alegre, a detecção tardia da DR-TB é um dos principais fatores agravantes da situação epidemiológica. Isso possivelmente se deve a falhas no sistema de saúde, pois os pacientes muitas vezes continuam a procurar os serviços de saúde até receberem um diagnóstico. Portanto, variáveis relacionadas ao comportamento dos pacientes e ao sistema de saúde contribuem para atrasos na detecção da DR-TB.

A idade média e a razão entre os sexos masculino e feminino observadas no presente estudo foram semelhantes às descritas pela Secretaria Municipal de Saúde de Porto Alegre, bem como às relatadas na literatura nacional e internacional.^(^ ^20^ ^,^ ^24^ ^-^ ^26^ ^)^ Também foi encontrado um número elevado de pacientes infectados pelo HIV na amostra. Isso talvez seja explicado pelos tipos de serviços de saúde investigados no presente estudo. É possível que alguns desses serviços tivessem equipes multidisciplinares e tratassem pacientes encaminhados por outros serviços de saúde. Constatamos também que os pacientes com história de tratamento antituberculose tinham maior probabilidade de terem sido testados para HIV, provavelmente porque procuraram serviços de diagnóstico e tratamento por meio de programas de controle da tuberculose nos quais o teste de HIV tornou-se um requisito de rotina.

As respostas para as perguntas de triagem sobre história de tratamento antituberculose indicaram que 61% dos pacientes não tratados previamente haviam sido previamente submetidos à radiografia de tórax, embora o Ministério da Saúde do Brasil não recomende a triagem radiográfica em pacientes com tosse produtiva e suspeita de tuberculose.^(^ ^13^ ^)^ No presente estudo, a história de sintomas de tuberculose foi investigada por meio de questões relacionadas a hemoptise, dor torácica e outros sintomas de tuberculose pulmonar. Esses sintomas foram identificados em 45% da amostra estudada e foram mais frequentes nos pacientes com história de tratamento antituberculose, como era de se esperar. Vale ressaltar que também investigamos hemoptise, a qual é um sintoma menos frequente que se apresenta mais tardiamente no curso da doença.^(^ ^27^ ^)^

A frequência de infecção pelo HIV entre os sujeitos do nosso estudo foi elevada mas menor do que a relatada no Sistema Nacional de Informação de Agravos de Notificação para Porto Alegre.^(^ ^8^ ^)^ Nossos resultados diferiram dos relatados na literatura, pois a incidência de DR-TB nos pacientes infectados pelo HIV com história de tratamento antituberculose foi maior em nossa amostra (32,8%). Um estudo realizado no estado de Santa Catarina (também no Sul do Brasil) constatou que a prevalência de infecção pelo HIV foi maior nos pacientes que nunca haviam sido tratados para tuberculose do que naqueles com história de tratamento antituberculose (20% vs. 9%).^(^ ^28^ ^)^ Nossos achados também reforçam a hipótese de que a frequência de DR-TB é maior em regiões onde há altas taxas de abandono do tratamento.

Os resultados do presente estudo mostram que é preciso a conscientização de autoridades, gestores e profissionais de saúde sobre estratégias de controle da tuberculose a fim de melhorar a situação de saúde na região estudada. Há uma necessidade urgente de aumentar a cobertura do tratamento, reduzir a taxa de abandono do tratamento e identificar estratégias para o diagnóstico precoce da DR-TB e da TBMR nas unidades básicas de saúde e nos hospitais de Porto Alegre. Estratégias eficazes poderiam incluir novos testes diagnósticos (cultura líquida ou teste molecular) ou o uso de regras de predição clínica. Esse último método foi sugerido por pesquisadores do Peru, um país com alta prevalência de DR-TB, onde significativos esforços técnicos e políticos têm sido feitos para a implantação de programas para o controle da DR-TB e da TBMR.^(^ ^29^ ^)^

No presente estudo, foi possível analisar o comportamento epidemiológico da DR-TB e as variáveis associadas a essa condição em um grupo de pacientes incluídos no II Inquérito Nacional de Resistência aos Fármacos Antituberculose, que foi realizado em Porto Alegre. O maior tempo entre o início dos sintomas e o diagnóstico de tuberculose e a história de tratamento antituberculose mostraram associação com a ocorrência de DR-TB e de TBMR. Caso esses resultados sejam confirmados em outros estudos no Brasil, essas variáveis poderão ser utilizadas como preditores de TBMR, contribuindo assim para a investigação e implantação da terapia medicamentosa adequada. Além disso, esses achados poderão promover menores taxas de morbidade e mortalidade, bem como reduzir o risco de transmissão da tuberculose na comunidade.
